# A Retrospective Risk-Factor Analysis of Patients Presenting With Peripheral Vascular Disease in a Tertiary Care Hospital in North-East India

**DOI:** 10.7759/cureus.82661

**Published:** 2025-04-20

**Authors:** Ranendra Hajong, Pinky Rabha, Bijit B Medhi, Shivalika Sharma, Pooja S Pai, Arup J Baruah, Khumanthem M Devi

**Affiliations:** 1 Surgery, North Eastern Indira Gandhi Regional Institute of Health and Medical Sciences, Shillong, IND; 2 General Surgery, North Eastern Indira Gandhi Regional Institute of Health and Medical Sciences, Shillong, IND; 3 General Surgery, Gauhati Medical College and Hospital, Guwahati, IND; 4 Transfusion Medicine, North Eastern Indira Gandhi Regional Institute of Health and Medical Sciences, Shillong, IND

**Keywords:** alcohol consumption, diabetes mellitus, duration of smoking, hypertension, north-east india, peripheral arterial disease, peripheral vascular disease, retrospective study, risk factors, smoking

## Abstract

Background

Peripheral vascular disease (PVD) encompasses both upper and lower limb diseases affecting veins and arteries. This study focuses on peripheral arterial disease (PAD) due to its higher morbidity and mortality rates related to cardiovascular deaths. Research is crucial to assess the prevalence, risk factors, and burden of PVD in the Indian population. There is a significant lack of data on PVD in the northeastern region of India; therefore, this study aims to examine the risk factors for PVD in patients attending a tertiary care hospital in Northeast India.

Methods

This hospital-based case-control study, conducted in both retrospective and prospective modes, aimed to identify risk factors in patients with PVD. The study was carried out in a tertiary care teaching hospital in Northeast India from January 2015 to January 2025. The cases were patients with PVD (Group A), and the controls were healthy volunteers without PVD (Group B). The study extended over a period of 10 years (January 2015-January 2025) and included retrospective data (January 2015-October 2020) from hospital records and prospective data (October 2020-January 2025) from a questionnaire survey and follow-ups, analysing disease progression and treatment outcomes.

Using a structured proforma, data were collected on sociodemographic details, symptoms, co-morbidities, substance misuse, and other relevant factors. The data was analyzed using IBM SPSS Statistics for Windows, Version 23.0 (Released 2015; IBM Corp., Armonk, New York, United States).

Results

A total of 172 cases and 688 controls, in a 1:4 ratio, participated in the study. Hypertension, diabetes mellitus, smoking history, and cardiac ailments were significantly higher among cases than controls (p<0.05). Among smokers, the number of cigarettes per day and smoking duration were significantly associated with peripheral vascular disease. When smoking was combined with diabetes mellitus and hypertension, the risk of developing peripheral vascular disease increased significantly. Atherosclerosis below the aortic bifurcation was found in 104 patients (60.47%), making it the most common vascular pathology, followed by thromboangiitis obliterans in 63 patients (36.63%).

Multivariate logistic regression analysis identified male gender (OR: 1.27, 95%CI: 0.019 to 0.231, p=0.01), smoking history (OR: 1.91, 95%CI: 0.638 to 0.972, p=0.001), number of cigarettes smoked per day (OR: 1.64, 95%CI: 0.473 to 0.816, p=0.002), diabetes mellitus (OR: 3.68, 95%CI: 0.488 to 1.368, p=0.000), and hypertension (OR: 2.17, 95%CI: 0.798 to 1.131, p=0.000) as independent risk factors for peripheral vascular disease. Amputation was the most common treatment offered, followed by rehabilitation. Bone marrow cell therapy was attempted in 21 patients, avoiding amputation in 17.

Conclusion

This result demonstrates smoking, diabetes mellitus, and hypertension as region-specific independent risk factors for PVD in Northeast India. These findings focus on the importance of reducing risk factors in patients with diabetes for the prevention of PVD.

## Introduction

Peripheral vascular disease (PVD) refers to obstructive disease of major vessels below the aortic bifurcation. Peripheral arterial disease (PAD) is associated with increased morbidity and a higher mortality rate due to cardiovascular-related deaths [1-3]. Lower extremity PAD is the third leading cause of atherosclerotic cardiovascular morbidity, after coronary artery disease and stroke [4]. The combined prevalence of PAD among individuals with type 2 diabetes mellitus across nine Indian states is estimated at 18% [5]. Hence, PVD is a global health problem with high morbidity and mortality [6]. A meta-analysis conducted by Fowkes et al. found that a conservative estimate of >202 million people were afflicted with PAD globally and also showed a relative increase in peripheral arterial disease prevalence of 23.5% during the first decade of the 21st century [4]. The prevalence of peripheral arterial disease among those aged 40-70 years and those over 70 years is 3-10% and 10-20%, respectively [7-10]. Age-adjusted prevalence of peripheral arterial disease in the South Indian population was found to be 26.7% [11].

PVD is linked to various modifiable and non-modifiable risk factors. Modifiable risk factors include diabetes mellitus [12], hypertension, and smoking [13], which can be managed through lifestyle changes and medical interventions. While PVD is associated with an increased risk of cardiovascular and cerebrovascular events [9,14-16], it is also a major risk factor associated with lower extremity symptoms, disability, and limb loss. Inflammation associated with diabetes plays a crucial role in PAD, serving both as a risk marker and as a potential risk factor that contributes to the development of PAD by damaging blood vessels, promoting plaque build-up, and increasing the risk of clot formation [12,17]. The combined prevalence of PAD in individuals with type 2 diabetes mellitus across nine Indian states is estimated to be 18% [5]. Diabetic patients with PVD are at a greater risk for lower limb amputation when compared with non-diabetic patients with PVD [18]. Non-modifiable risk factors such as male gender [9] and increasing age [19] cannot be altered but contribute significantly to disease susceptibility.

Studies on risk factors for PVD are mostly derived from Western countries, with limited data available in developing countries like India [20,21]. Hence, this study was designed to investigate the various risk factors most commonly associated with peripheral vascular disease in a tertiary care hospital of northeast India.

## Materials and methods

This case-control study was conducted in a tertiary care teaching hospital, North East Regional Institute of Health and Medical Sciences (NEIGRIHMS), Shillong, Meghalaya, in Northeast India to study the risk factors in patients with PVD. This study was conducted over 10 years, from January 2015 to January 2025. The study combined retrospective and prospective approaches to analyse disease progression and treatment outcomes and was approved by the Institutional Ethics Committee, NEIGRIHMS (approval number: NEIGR/IEC/M12/F13/2020).

Participants

A total of 172 cases treated for PVD during the study period were included in the study. Out of these, 78 patients were from the retrospective data (2015-2020), and 94 patients were from the prospective group (2020-2025). The control group consisted of healthy individuals without PVD, selected based on the same criteria where applicable, and who also provided informed consent to participate. For each case of PVD, four controls were included, consisting of healthy volunteers with no signs or symptoms of peripheral vascular disease. Hence, a total of 688 controls were included in the study after obtaining informed consent from the participants. Unwilling patients and patients with post-traumatic gangrene of limbs, malignant ulcers, and venous ulcers were excluded from the study.

Data collection

Retrospective data from 2015 to 2020 were collected from patient records of all individuals treated for PVD during that time, in accordance with the study’s predefined inclusion and exclusion criteria.

From 2020 to 2025, patients with PVD who met the eligibility criteria and provided informed consent were prospectively enrolled in the study. A validated questionnaire proforma (see Appendices) was designed for the study, which included a sociodemographic profile, detailed history regarding symptomatology amongst cases (intermittent claudication, rest pain, and gangrene) and presence of comorbidities (diabetes, hypertension, cardiac ailments, cerebrovascular accidents), history of substance misuse, was obtained from both prospective cases and controls. Routine blood investigations, ankle brachial pressure index (ABPI), colour doppler scanning of both lower limbs arterial system, and CT angiography were performed in the patients with PVD. The various treatment modalities offered to the patients were also recorded.

Statistical analysis

Statistical analysis of the data was done using IBM SPSS Statistics for Windows, Version 23.0 (Released 2015; IBM Corp., Armonk, New York, United States). Categorical variables are presented as frequencies and percentages. Continuous variables are presented as mean and standard deviation (SD). The characteristics of the study population were compared between the cases with PVD and healthy controls. The comparison of continuous variables between the groups was done by a two-sample t-test. The χ2 test was employed to compare the distribution of dichotomous variables between cases and controls. The multivariate logistic regression analysis was used to identify the independent risk of each variable with PVD, adjusted by confounders. To further explore significant independent risk factors for PVD, a backward selection algorithm was used. The association between the prevalence of PVD with independent risk factors was explored with adjustment for other risk factors. A p-value of <0.05 was considered statistically significant.

## Results

A total of 172 cases (98 male and 74 female) and 688 controls (368 male and 320 female) participated in the study. The mean age was 41.24 ± 7.63 years in the case group and 43.65 ± 8.29 years in the control group, with no statistically significant difference. All patient-related comparisons of sociodemographic factors, risk factors, and clinical characteristics are presented in Table 1. Among patients with PVD, 74.42% were smokers, 56.98% had hypertension, 38.37% had diabetes, and 15.12% had cardiac ailments, compared to 56.10%, 29.22%, 10.61%, and 3.92%, respectively, in the control group, differences that were statistically significant.

**Table 1:** Characteristics of cases and controls P values were calculated from χ2 test for categorical variables or two-sample t-test for continuous variables

^Parameters^	Group A (n=172), n (%)	Group B (n=688), n (%)	P value	Chi square/t value
Male gender	98 (56.98%)	368 (53.49%)	0.097	9.378
Mean age (years)	41.24 ± 7.63	43.65 ± 8.29	0.03*	0.278
Hypertension	98 (56.98%)	201 (29.22%)	< .001^*^	13.978
Diabetes Mellitus	66 (38.37%)	73 (10.61%)	0.046*	66.526
Cardiac ailments	26 (15.12%)	27(3.92%)	0.034*	21.221
Smoking	128 (74.42%)	386 (56.10%)	0.007*	188.577
Smoking duration (years)			< .001*	198.819
0-10	15 (11.72%)	158(40.93%)		
11-20	36 (28.13%)	141(36.53%)		
21-30	51 (39.84%)	54 (13.99%)		
31-40	26 (20.31%)	33 (8.55%)		
Number of cigarettes per day			0.001*	190.716
1-5	14 (10.94%)	109 (28.24%)		
6-10	27 (21.09%)	162 (41.97%)		
11-15	40 (31.25%)	76 (19.69%)		
16-20	33 (25.78%)	28 (7.25%)		
>20	14 (10.94%)	11 (2.85%)		
Alcohol	33 (19.18%)	121(17.59%)	0.54	54.11
Diabetes mellitus + Smoking	54 (31.40%)	69 (10.03%)	< .001*	62.659
Diabetes mellitus + Hypertension	57 (33.14%)	66 (9.59%)	0.01*	59.132
Smoking + Hypertension	91 (52.91%)	177 (25.73%)	0.002*	15.176

The lower limb was exclusively involved in patients with PVD. The most common presentation in patients with PVD was gangrene in its various forms. Dry gangrene was detected in 57 (33.14%) patients. Atherosclerotic changes of the aorta below the level of bifurcation were detected in 104 (60.47%) patients with PVD. Thromboangiitis obliterans was diagnosed in 63 (36.63%) patients, either by CT angiography, colour Doppler scanning, or at biopsy after amputation. The mean ABPI in patients with PVD was 0.71 ± 0.13. Bilateral involvement of the lower limb was detected in 29 patients (16.86%) at a median duration of 7.24 ± 2.94 years. The level of involvement in patients with PVD was as follows: forefoot involvement in 27 patients (15.70%), up to the ankle joint in 53 patients (30.81%), below-knee involvement in 68 patients (39.53%), and above-knee involvement in 24 patients (13.95%).

Amputation was the most common modality of treatment offered to the patients. A total of 141 (81.98%) patients underwent amputation followed by rehabilitation. Bone marrow cell therapy was tried in 21 (12.21%) patients with critical limb ischemia, with signs of improvement in 17 (80.95%) patients, avoiding amputation.

The risk of developing PVD was found highest amongst diabetics (OR: 5.246, 95%CI: 3.546-7.760), followed by cardiac ailments (OR: 4.360, 95%CI: 2.472-7.691), hypertension (OR: 3.208, 95%CI: 2.276-4.525), and smoking (OR: 2.276, 95%CI: 1.566-3.309) as mentioned in Table 2. Amongst smokers, it was the number of cigarettes smoked per day and the duration of smoking that determined their risk of developing PVD.

**Table 2:** Association between risk factors and prevalence of peripheral artery disease

Risk factor		Odds ratio	95%CI	p value
Hypertensive		3.208	2.276 to 4.525	< .001*
Smoking		2.276	1.566 to 3.309	< .001*
Smoking duration (years)	0-10	Reference category	Reference category	< .001*
	11-20	1.027	0.681 to 1.550	
	21-30	4.949	3.221 to 7.602	
	31-40	3.535	2.051 to 6.092	
Number of cigarettes per day	0-5	Reference category	Reference category	0.001*
	6-10	0.605	0.387 to 0.945	
	11-15	2.44	1.593 to 3.738	
	16-20	5.596	3.275 to 9.563	
	>20	5.453	2.430 to 12.240	
Diabetes mellitus		5.246	3.546 to 7.760	< .001*
Cardiac ailment		4.36	2.472 to 7.691	< .001*
Alcohol		1.113	0.726 to 1.706	0.63
Diabetes mellitus + Smoking		3.13	2.285 to 4.287	< .001*
Diabetes mellitus + Hypertension		3.455	2.527 to 4.722	< .001*
Smoking + Hypertension		2.057	1.701 to 2.486	< .001*

Backward multivariate regression analyses of risk factors for peripheral vascular disease are shown in Table 3. Male sex (OR: 1.27, 95%CI: 0.019-0.231, p value: 0.010), history of smoking(OR: 1.91, 95%CI: 0.683-0.972, p value: 0.001), number of cigarettes smoked per day (OR: 1.64, 95%CI: 0.473-0.816, p value: 0.002), diabetes mellitus (OR: 3.68, 95%CI: 0.488-1.368, p value: 0.000), and participants with diabetes mellitus who were smokers (OR: 2.43, 95%CI: 0.577-0.944, p value: 0.000) were associated with significantly higher prevalence of PVD and were found to be the independent risk factor associated with PVD (Table 3).

**Table 3:** Multivariate regression analysis of risk factors associated with peripheral vascular disease Logistic regression model adjusted for sex, age (continuous) ^b^For ordinal variables, the p-value was estimated from the linear trend test

Risk factor	Odds ratio	95%CI	p value ^b^
Male gender	1.27	0.019 to 0.231	0.010*
Age	1.02	0.261 to 0.279	0.440
Smoking	1.91	0.638 to 0.972	0.001*
Smoking duration	1.32	0.071 to 0.213	0.020*
No of cigarettes per day	1.64	0.473 to 0.816	0.002*
Alcohol	1.01	0.096 to 0.103	0.090
Hypertension	2.17	0.798 to 1.131	˂0.001*
Diabetes mellitus	3.68	0.488 to 1.368	˂0.001*
Diabetes mellitus + smoking	2.43	0.577 to 0.944	˂0.001*
Diabetes mellitus + Hypertension	2.51	0.683 to 1.013	˂0.001*
Smoking + Hypertension	2.75	0.871 to 0.946	0.001*

## Discussion

This is the first case-control study on risk factor analysis of PVD in Northeast India, to the best of our knowledge. By identifying the region-specific risk factors for PVD, preventive measures can be addressed. By multivariate analysis, the presence of a history of smoking, diabetes mellitus, smoking with diabetes mellitus, and hypertension were found to be independent risk factors for PVD among the population in Northeast India. Among the risk factors analysed, diabetes mellitus showed the strongest association with PVD. This is consistent with the Framingham Heart Study and its subsequent studies, which have reported a strong association between diabetes and PVD [22,23]. Diabetes mellitus was also reported to be the strongest risk factor by the American Diabetes Association [12]. In a study done in Sri Lanka, diabetes mellitus, hypertension, or dyslipidaemia for 10 years, elevated CRP (C-reactive protein), and hyperhomocysteinemia were found to be independent risk factors for PVD [24]. A study done on the San Diego population also found the strongest association with diabetes mellitus (OR = 6.9) [25]. Dyslipidaemia was reported to be a significant risk factor associated with PVD according to Ridker et al. [26] and the National Health and Nutrition Survey [8]. In the present study, a significant association was observed between PVD and diabetes, as the OR of diabetes mellitus was 3.68 (p=0.000).

In multivariate regression analysis, smoking emerged as another strong risk factor for PVD. This was in concurrence with many previous studies, with a consistent dose-response relationship [13,27]. According to various studies, smoking in the past and present (OR 1.5, 95%CI: 1.1-2.1) is a significant risk factor for PVD [28,29]. However, in another study, it was found that a younger age at smoking initiation was associated with a higher risk of developing PVD [30]. Pack years of smoking of more than 10-20 years significantly increase the risk of developing PVD, as shown in the Framingham study [1] and the San Diego Population study [25].

In line with studies conducted in Finland [31], Rotterdam [19], and the San Diego population [25], our study also identified hypertension as a significant risk factor for PVD, with an OR of 2.17, demonstrating a strong association after multivariate analysis.

Alcohol consumption showed no association with PVD in the present study, in concurrence with a study done in Edinburgh [32], unlike a study done in the United States, which reported a negative association between alcohol intake and PVD [33]. However, in a recent meta-analysis, it was found that greater than or equal to 10 drinks/week is associated with increased risk of PVD [34].

Smoking, duration of smoking, and number of cigarettes smoked per day have significant associations as risk factors for PVD in patients with diabetes mellitus. Alzahrani et al. reported duration of diabetes and type of diabetes as significant risk factors [35].

A limitation of the study was the absence of routine ABPI screening and radiological investigations in the control group, which may have affected the accurate exclusion of PVD. Furthermore, this study was limited by both recall bias and the inability to determine the incidence and causality of PVD in this population.

The strength of the study lies in the fact that this is the first case-control study for the risk factors in the northeastern part of India, which will act as the baseline information for future research. The sample size of the cases and controls (four times the cases) adds to the credibility of the study.

## Conclusions

Smoking, diabetes mellitus, and alcohol consumption were identified as region-specific independent risk factors for PVD in Northeast India. These findings highlight the importance of reducing risk factors for the prevention of PVD. They also highlight the potential to identify patients with a high risk of PVD based on common risk factors, i.e., smoking. Further prospective cohort studies are recommended to address the prediction of PVD in Northeast India.

## Figures and Tables

**Figure 1:**
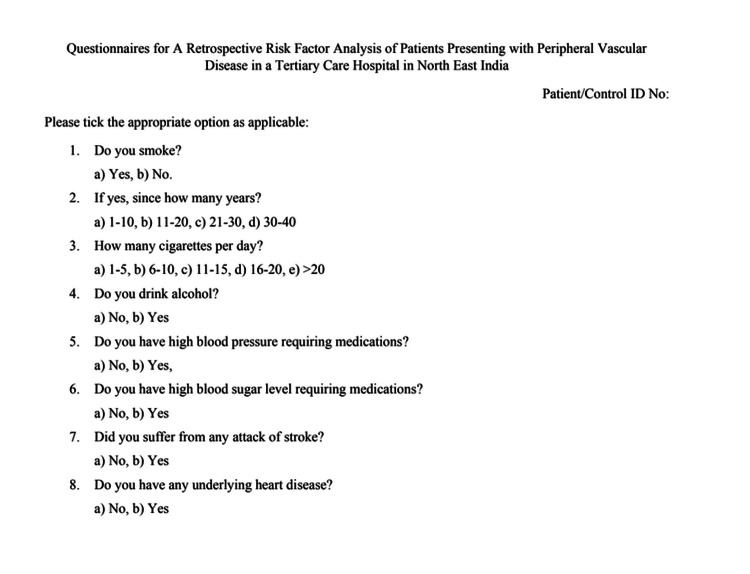
Questionnaire
